# Association of serum fibroblast growth factor 21 with diabetic complications and insulin dose in patients with type 1 diabetes mellitus

**DOI:** 10.1371/journal.pone.0263774

**Published:** 2022-02-22

**Authors:** Hirokazu Taniguchi, Shinsuke Nirengi, Kengo Ishihara, Naoki Sakane

**Affiliations:** 1 Division of Applied Life Sciences, Graduate School of Life and Environmental Sciences, Kyoto Prefectural University, Kyoto, Japan; 2 Faculty of Agriculture, Ryukoku University, Shiga, Japan; 3 Division of Preventive Medicine, Clinical Research Institute, National Hospital Organization Kyoto Medical Center, Kyoto, Japan; 4 Department of Physiology and Cell Biology, Dorothy M. Davis Heart and Lung Research Institute, The Ohio State University Wexner Medical Center, Columbus, Ohio, United States of America; Medical School, University of Zagreb, CROATIA

## Abstract

**Introduction:**

Fibroblast growth factor (FGF) 21 is an important regulator of glycemic control, but the association between circulating FGF21 and diabetic complications is poorly understood. Moreover, basal FGF21 secretion, especially in response to insulin dose, in patients with type 1 diabetes mellitus (T1DM), has not been well examined. Therefore, this study aimed to determine the association of circulating FGF21 levels with diabetic complications and insulin dosage in middle-aged and elderly patients with T1DM.

**Materials and methods:**

A total of 127 middle-aged and elderly patients with T1DM, including 68 patients with diabetic complications, and 106 non-diabetic individuals were analyzed in this cross-sectional study. Information on demographic characteristics and T1DM was extracted from their electronic medical records. Serum FGF21 levels were determined using ELISA.

**Results:**

Serum FGF21 levels were significantly lower in T1DM patients (75.2 [37.4–135.1] pg/mL) than in non-diabetic participants (151.6 [92.0–224.6] pg/mL; P < 0.001). No diabetic complications were associated with serum FGF21 concentrations. Both basal and bolus insulin doses were significantly and positively correlated with serum FGF21 levels (P < 0.05). Stepwise multiple regression analysis showed that FGF21 level was associated with age and body mass index (P < 0.05), while the basal insulin dose was an independent positive predictor of serum FGF21 levels (β = 0.197, P = 0.032).

**Conclusions:**

Circulating FGF21 levels are reduced in patients with T1DM; however, they are not associated with diabetic complications. In addition, aging, obesity, and insulin dosage are positive determinants of circulating FGF21.

## Introduction

Fibroblast growth factor (FGF) 21 induces energy expenditure [1] and glucose uptake in adipocytes and myotubes [2, 3]. In addition, administration of FGF21 and its analog has beneficial effects on glycemic control in mice with both type 1 and 2 diabetes models [2, 4] and in humans with type 2 diabetes mellitus [5]. These results suggest that FGF21 plays a significant role in glucose homeostasis in diabetic patients.

Since FGF21 is highly expressed in the pancreas [6], it is possibly a secretory organ for FGF21. Serum FGF21 levels were found to be lower in mice with streptozotocin-induced type 1 diabetes [7] and in patients with type 1 diabetes mellitus (T1DM) [8–10]. T1DM is characterized by pancreatic beta-cell destruction, and thus, it is plausible that this dysfunction attenuates FGF21 secretion from the pancreas. Decreased FGF21 secretion may lead to poor glycemic control and diabetic complications. However, the relationship between circulating FGF21 levels and other diabetic complications has not been studied because of the lower incidence of T1DM.

It was previously reported that FGF21 secretion was increased by glucose intake [11] and that insulin was responsible for the rise in circulating FGF21 levels in adult humans, as detected by the hyperinsulinemic-euglycemic clamp [12]. These results suggest that insulin secretion from pancreatic beta cells plays a regulatory role in maintaining the concentration of circulating FGF21. Thus, lower levels of serum FGF21 in T1DM might be caused by the depletion of insulin stimulation instead of pancreatic dysfunction. To maintain blood glucose levels, basal insulin is continuously released from the pancreas and it increases after every meal intake. Previous results suggest that, because basal and postprandial administration of bolus insulin is part of insulin therapy in patients with T1DM [13], FGF21 secretion may be therefore, influenced by changes in both dosage levels.

In our study, we identified how insulin doses and diabetic complications are related to circulating FGF21 levels in patients with T1DM. The results were significant because they show whether lower FGF21 levels due to lower insulin secretion from the pancreas lead to diabetic complications and that the sensitivity of FGF21 to insulin is independent of pancreatic beta-cell failure.

## Materials and methods

### Study design and subjects

**Fig 1:**
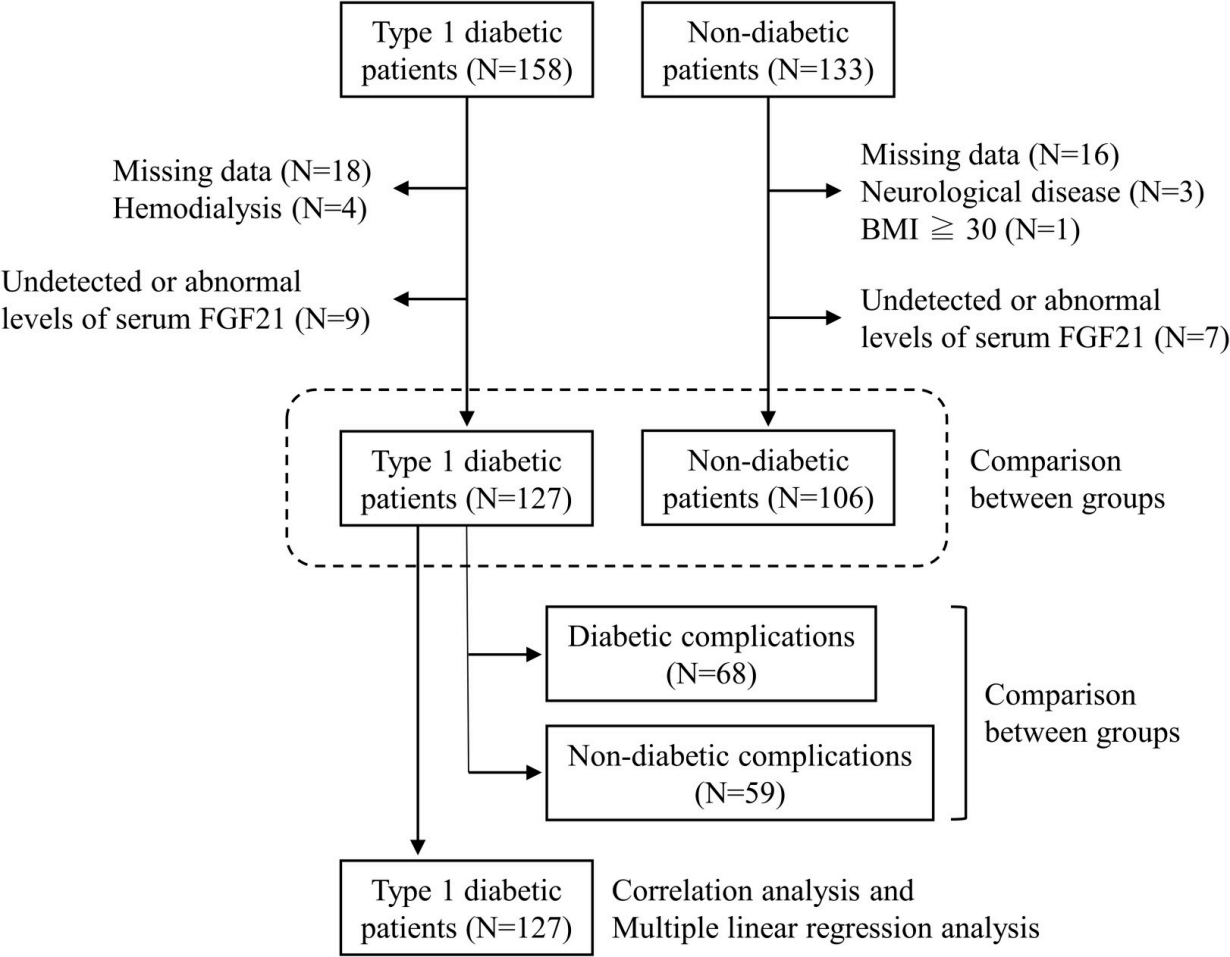
Flow diagram of participants in the cross-sectional study.

As shown in Fig 1, 158 Japanese patients with T1DM participated in this cross-sectional study. These patients visited the National Hospital Organization, Kyoto Medical Center and they were middle-aged or older (aged 40–79 years). Thirty-one T1DM patients were excluded from the analysis because of missing data, hemodialysis, and undetectable or abnormal serum FGF21 levels. Serum FGF21 levels and T1DM characteristics were compared between patients with and without diabetic complications. Correlation analysis and multiple linear regression analysis were used to examine the determinant factors of circulating FGF21 levels in patients with T1DM.

Middle-aged and elderly (aged 40–79 years), 133 non-diabetic individuals were included to investigate whether serum FGF21 levels were lower in T1DM patients than in non-diabetic individuals. These non-diabetic individuals were ambulatory patients at the Kyoto Medical Center and were selected from those with hyper low-density lipoprotein (LDL) cholesterolemia only. This was because serum LDL cholesterol was found to be not associated with serum FGF21 levels as described previously [14–16]. Twenty-seven of these subjects were excluded from the analysis on account of missing data, neurological disease, obesity, and undetectable or abnormal levels of serum FGF21. Therefore, finally 127 T1DM patients and 106 non-diabetic individuals were successfully analyzed in this study.

All participants provided written informed consent to participate before enrolling in the study, which was approved by the Ethics Committee of Kyoto Medical Center (approval number: 09–036 and 15–093). The study was conducted in accordance with the principles of the Declaration of Helsinki.

### Diagnosis and data collection

T1DM was diagnosed based on the criteria of the Japan Diabetes Society [17]. A target level of hemoglobin A1c (HbA1c) < 8% was used as “Objective when intensification of therapy considered difficult” and that of < 7% was used as “Objective when aiming to prevent complications” in accordance with the guidelines of the Japan Diabetes Society [18]. Information on demographic characteristics (sex, age, height, weight, body mass index [BMI], systolic blood pressure [SBP], diastolic blood pressure, alcohol consumption, and smoking), and T1DM related information (diabetic complications, duration of diabetes, and insulin dose) were extracted from the patients’ electronic medical records.

### Analysis of blood samples

Blood samples were collected in the morning to analyze aspartate aminotransferase (AST), alanine aminotransferase (ALT), γ-glutamyl transpeptidase (γ-GTP), triglyceride (TG), creatinine (CRE), and glucose levels. Blood biomarkers were measured using an automatic biochemical analyzer (AU580; Beckman Coulter, Inc., Tokyo, Japan). Estimated glomerular filtration rate (eGFR) was calculated using the following equation: 194 × Cr^-1094^ × age^-0.287^ [19]. HbA1c levels were measured using an ADAMS A1c HA-8180 automatic glycohemoglobin analyzer (Arkray Inc., Kyoto, Japan).

The serum FGF21 concentration was determined using a commercially available ELISA kit (DF2100; R&D Systems, Inc.) according to the manufacturer’s instructions. The mean minimum detectable dose of the assay was 4.67 pg/mL. The intra- and inter-assay coefficients of variation reported by the manufacturer were 2.9%–3.9% and 5.2%–10.9%, respectively.

### Statistical analysis

All statistical analyses were performed using SPSS (version 24.0; SPSS, Inc.). The Kolmogorov-Smirnov test was performed to assess the normality of the data distribution, and several variables were log-transformed prior to analysis. The Mann-Whitney U test (for distributed data) and the chi-square test (for categorical data) were used to evaluate the differences among the non-diabetic and T1DM patients with and without diabetic complications. Associations among the variables were detected using Spearman’s correlation coefficients. Partial correlation analysis was performed after adjusting for sex and age.

We performed stepwise multiple linear regression analysis to identify factors influencing FGF21 expression in T1DM patients. Since sex, age, BMI, SBP, TG, basal insulin dose, and bolus insulin dose showed significant partial correlation with FGF21 after adjustment for sex and age, these variables were included in the linear regression model. All measurements and calculated values are presented as the mean ± standard deviation (SD) (for normally distributed variables) or as the median (interquartile ratio for non-normally distributed variables). The level of statistical significance was set at P < 0.05.

## Results

### Comparison of the characteristics between T1DM and non-diabetic patients

The patient characteristics are shown in Table 1. There were no statistically significant differences in sex, age, BMI, blood pressure, drinking, and smoking habits between the two groups. Blood glucose and HbA1c levels were significantly higher in T1DM patients than in non-diabetic patients (P < 0.001), while other blood parameters such as AST, ALT, γ-GTP, CRE, and eGFR did not differ between the groups. Average onset of T1DM in the patients was 43.4 ± 16.0 years, and the average disease duration was 15.0 (8.0–22.0) years. Diabetic complications were observed in 68 patients (53.5%).

**Table 1:** Characteristics of the type 1 diabetic and non-diabetic patients.

Variable	Type 1 diabetes	Non-diabetes	P
N	127	106	
Sex, M/F	47/80	32/74	0.275
Age, y	59.0 (47.0–69.0)	63.0 (52.0–69.0)	0.308
Height, cm	161±8	159 (154–164)	0.086
Weight, kg	57.2 (52.0–61.9)	55.0 (48.7–63.8)	0.277
BMI, kg/m^2^	22.2±2.9	22.2±2.9	0.854
SBP, mmHg	127 (120–136)	129±18	0.875
DBP, mmHg	73.8±10.1	76.9±12.8	0.054
Alcohol intake, %	25.2	35.8	0.085
Current smoking, %	17.3	9.4	0.078
Blood chemical analysis			
AST, IU/L	21.0 (18.0–26.0)	22.0 (20.0–26.0)	0.211
ALT, IU/L	17.0 (14.0–22.0)	19.0 (14.8–23.3)	0.145
γ-GTP, IU/L	20.0 (13.0–30.0)	21.0 (15.0–26.0)	0.791
CRE, mg/dL	0.70 (0.62–0.85)	0.71 (0.62–0.84)	0.789
eGFR, ml/min/1.73m^2^	72.4 (61.0–83.2)	69.4±15.4	0.101
TG, mg/dL	86.0 (60.0–121.0)	92.7±26.7	0.355
Glucose, mg/dL	176.0 (126.0–211.0)	96.0 (90.0–105.0)	**< 0.001**
HbA1c, %	7.8 (7.3–8.4)	5.7 (5.6–6.0)	**< 0.001**
Diabetes history			
Age at onset, y	43.4±16.0		
Duration of diabetes, y	15.0 (8.0–22.0)		
Basal insulin dose, IU/day	10.0 (6.5–14.0)		
Bolus insulin dose, IU/day	19.5 (15.5–26.8)		
Diabetic retinopathy, %	26.8		
Diabetic nephropathy, %	25.2		
Diabetic neuropathy, %	22.0		

Data are the mean ± SD or median (interquartile ratio) values. Boldface indicates significance.

### Distribution of the serum FGF21 levels in T1DM and non-diabetic patients

**Fig 2:**
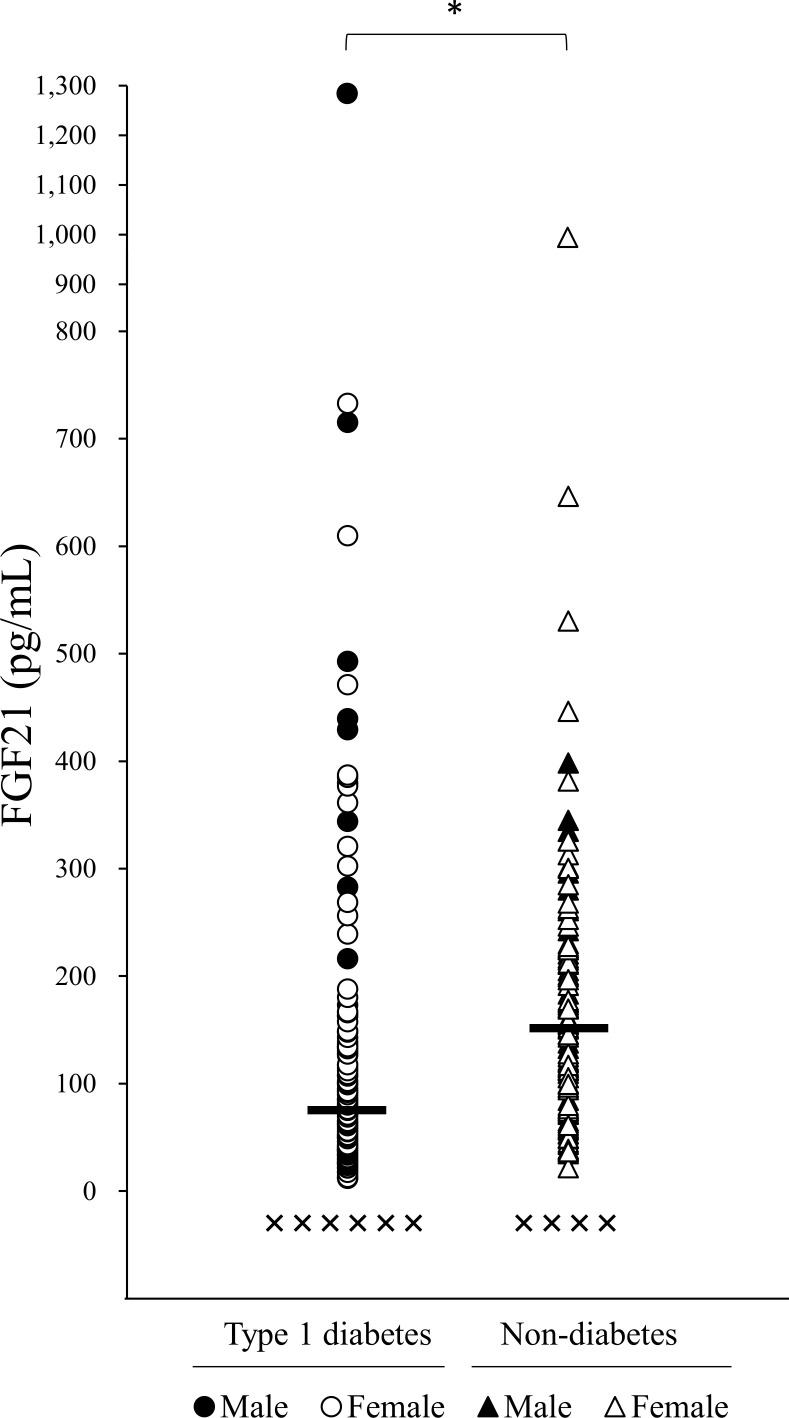
Distribution of the serum FGF21 concentration in T1DM and non-diabetic middle-aged and elderly subjects. Closed circles (●) indicate male T1DM patients, and open circles (○) indicate female T1DM patients. Closed triangles (▲) indicate male non-diabetic participants and open triangles (△) indicate female non-diabetic participants. Cross mark (×) shows undetectable serum FGF21 levels in each group. Horizontal lines represent the median values.

Six patients with T1DM and four non-diabetic subjects had undetectable levels of serum FGF21 (Fig 2). The upper range (≥ 2000 pg/mL) levels of serum FGF21 were observed in three participants from each group. The average serum FGF21 level in T1DM patients was 75.2 (37.4–135.1) pg/mL, which was significantly lower than that in the non-diabetic group (151.6 [92.0–224.6] pg/mL; P < 0.001).

### Effects of diabetic complications on the serum FGF21 level in T1DM patients

The variables in T1DM patients with and without diabetic complications were compared accordingly (Table 2). This analysis included 68 patients with diabetic complications (34 retinopathies, 32 nephropathies, and 28 neuropathies) and 59 patients with non-diabetic complications. The prevalence of multiple complications was 18.1% in all patients with T1DM (n = 23).

**Table 2:** Characteristics of type 1 diabetic patients with and without diabetic complications.

Variable	Diabetic Retinopathy	Diabetic Nephropathy	Diabetic Neuropathy	Diabetic Complications	Non-diabetic Complications
N	34	32	28	68	59
Sex, M/F	14 / 20	13 / 19	11 / 17	26 / 42	21 / 38
Age, y	60.5±12.9	62.6±11.6*	60.0±13.5	64.5 (50.3–70.0)*	53.0 (45.0–68.0)
Height, cm	162±9	162±9	161±10	161±9	161±7
Weight, kg	60.2 (55.0–64.9)*	59.3±11.5	59.6±11.3	58.8±10.5	56.6±8.2
BMI, kg/m^2^	22.3 (21.1–25.3)*	22.5±3.5	22.9±3.2	22.3 (20.6–24.2)	21.8±2.5
SBP, mmHg	129±14	130±14	129±13	128±13	128 (120–134)
DBP, mmHg	73.5±11.5	73.4±9.6	74.8±11.7	73.4±10.2	74.2±9.9
Alcohol intake, %	17.6	28.1	21.4	19.1	32.2
Current smoking, %	11.8	25.0	21.4	19.1	15.3
Blood chemical analysis					
AST, IU/L	21.0 (18.8–28.0)	21.5 (20.0–27.5)*	21.0 (19.0–32.0)	21.0 (19.0–28.8)*	20.0 (17.0–24.0)
ALT, IU/L	18.0 (14.8–24.5)*	18.0 (15.0–27.0)*	18.5 (15.0–30.3)*	18.0 (15.0–26.8)*	16.5±5.6
γ-GTP, IU/L	22.5 (14.8–44.8)*	20.5 (14.5–46.3)*	22.5 (16.0–39.5)*	20.5 (15.0–37.5)*	16.0 (12.0–26.0)
CRE, mg/dL	0.77 (0.66–0.90)*	0.72 (0.65–1.06)*	0.69 (0.62–0.86)	0.73 (0.63–0.94)*	0.69 (0.59–0.76)
eGFR, ml/min/1.73m^2^	70.0±20.3	66.3±18.2*	69.5 (60.3–82.3)	70.5±23.7*	77.8±16.7
TG, mg/dL	84.0 (67.0–129.8)	86.5 (60.5–121.0)	120.0±60.1*	87.5 (67.0–127.5)	88.1±37.0
Glucose, mg/dL	182±63	177±69	178 (131–202)	174 (129–223)	177 (124–205)
HbA1c, %	8.2 (7.6–9.0)*	7.7 (7.3–8.6)	8.5±1.5*	8.1 (7.4–8.9)*	7.5 (7.1–8.1)
FGF21, pg/mL	76.3 (34.0–148.0)	82.5 (39.1–141.4)	74.0 (40.6–137.9)	79.6 (39.1–142.0)	72.8 (34.5–130.3)
Diabetes history					
Age at onset, y	37.4±14.0	44.9±14.1	45.1±17.8	43.5±16.2	43.2±15.9
Duration of diabetes, y	23.1±11.0*	17.7±11.6	14.9±8.5	18.0±11.0*	12.0 (7.0–19.0)
Basal insulin dose, IU/day	11.1 (8.0–16.3)	10.0 (6.3–15.5)	11.2±7.3	10.0 (6.6–15.0)	10.0 (6.0–14.0)
Bolus insulin dose, IU/day	19.5 (15.0–25.1)	19.2±9.3	18.0 (16.1–24.0)	18.5 (15.0–24.4)	20.0 (16.0–31.0)

Data are the mean ± SD or median (interquartile ratio) values. Boldface and * indicates significance compared with non-diabetic complications (P < 0.05).

Patients with diabetic complications showed significantly lower eGFR, higher age, AST, ALT, γ-GTP, CRE, HbA1c, and duration of diabetes compared to those with no diabetic complications (P < 0.05). Also, there was no significant difference in serum FGF21 level between patients with diabetic complications (79.6 [39.1–142.0] pg/mL) and those with non-diabetic complications (72.8 [34.5–130.3] pg/mL). This implies that diabetic complications were not associated with serum FGF21 concentrations (Table 2). Multiple complications were also not related to serum FGF21 levels (62.9 [38.1–140.9] pg/mL).

### Association of the serum FGF21 level with body composition and metabolic parameters in T1DM patients

Table 3 shows correlations between the serum FGF21 level and other variables. The single correlation analysis revealed that serum FGF21 level was significantly and positively correlated with age, BMI, SBP, γ-GTP, TG, and basal insulin dose (P < 0.01). eGFR was significantly and negatively associated with serum FGF21 concentration (P = 0.007).

**Table 3:** Correlations of the serum FGF21 level with the other variables in type 1 diabetic patients.

	FGF21		FGF21 (Sex and Age Adjusted)	
(N = 127)	Rho	P	Rho	P
Age, y	0.237	**0.007**		
BMI, kg/m^2^	0.304	**< 0.001**	0.296	**< 0.001**
SBP, mmHg	0.294	**< 0.001**	0.229	**0.010**
DBP, mmHg	0.104	0.246	0.120	0.182
AST, IU/L	0.130	0.146	0.070	0.435
ALT, IU/L	0.111	0.216	0.072	0.427
γ-GTP, IU/L	0.240	**0.007**	0.148	0.098
eGFR, ml/min/1.73m^2^	-0.236	**0.007**	-0.154	0.086
TG, mg/dL	0.253	**0.004**	0.199	**0.026**
Glucose, mg/dL	-0.005	0.959	-0.011	0.899
HbA1c, %	0.117	0.191	0.051	0.576
Duration of diabetes, y	-0.092	0.306	-0.114	0.206
Basal insulin dose, IU/day	0.228	**0.010**	0.250	**0.005**
Bolus insulin dose, IU/day	0.159	0.075	0.182	**0.042**

Data are the Spearman’s rank correlation coefficients. Boldface indicates significance.

**Fig 3:**
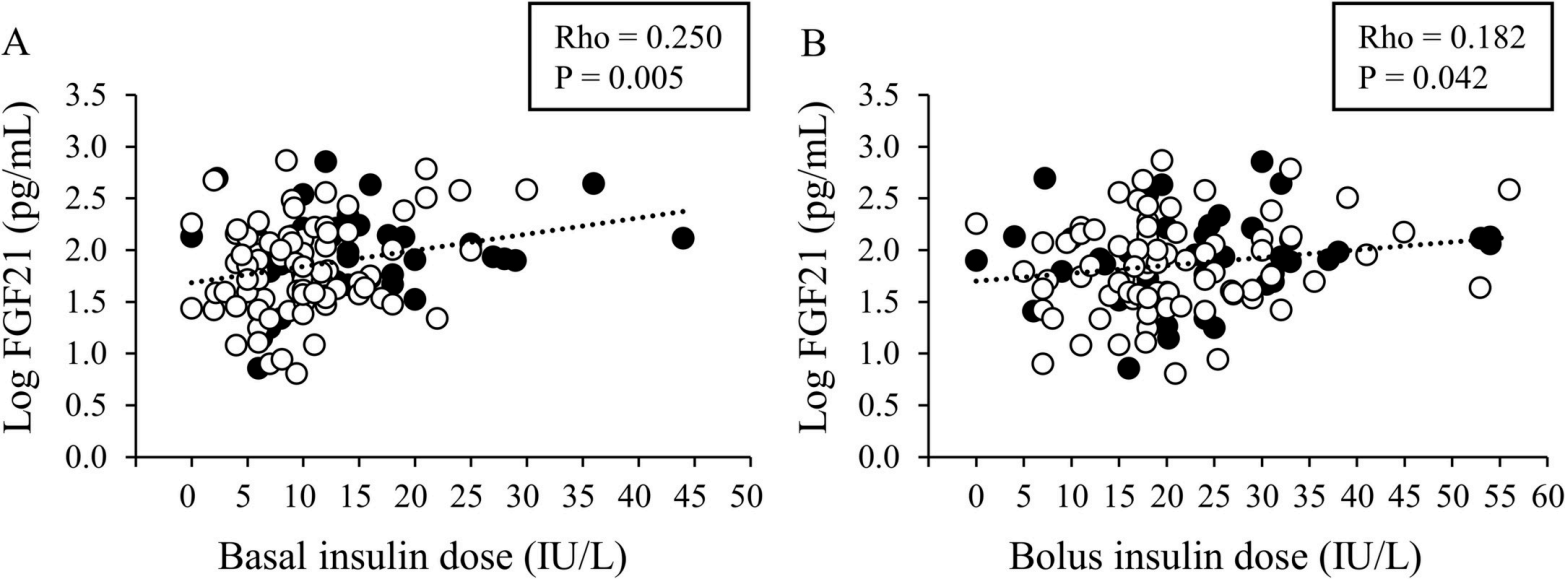
Correlation of the serum FGF21 level with basal insulin dose (A) and bolus insulin dose (B) in middle-aged and elderly T1DM patients. Closed circles (●) indicate male patients, and open circles (○) indicate female patients.

After adjusting for sex and age, serum FGF21 levels were still found to be significantly and positively correlated with BMI, SBP, TG, and basal insulin dose (P < 0.05). Although simple correlation analysis indicated that bolus insulin dose tended to be associated with serum FGF21 concentration (P = 0.075), the partial correlation analysis showed that serum FGF21 level was significantly associated with bolus insulin dose (P = 0.042) in comparison to the basal insulin dose (Fig 3).

### Determinant factors of the serum FGF21 concentration in T1DM patients

Stepwise multiple regression analysis was used to examine the independent predictors of serum FGF21 levels in patients with T1DM (Table 4). The analysis included sex, age, BMI, SBP, TG, basal insulin dose, and bolus insulin dose as independent variables. In the best-fitting model, serum FGF21 level was associated with age (β = 0.267, P = 0.002) and BMI (β = 0.211, P = 0.021), while the basal insulin dose was also an independent positive predictor of serum FGF21 levels (β = 0.197, P = 0.032).

**Table 4:** Stepwise multiple linear regression analysis of FGF21 in type 1 diabetes patients.

Dependent Variable	Independent Variable	β	P
FGF21, pg/mL	Age, y	0.267	**0.002**
	BMI, kg/m^2^	0.211	**0.021**
	Basal insulin dose, IU/day	0.197	**0.032**

N = 127. The model was adjusted for sex, SBP, TG, and bolus insulin dose. FGF21 and SBP was log transformed prior to analysis (model r^2^ = 0.165, P = 0.032). Boldface indicates significance.

## Discussion

The present study examined the determinant factors of circulating FGF21 concentration in middle-aged and elderly patients with T1DM. Cross-sectional analysis revealed that serum FGF21 levels were lower in T1DM patients than in non-diabetic individuals; however, no diabetic complications contributed to serum FGF21 concentration. In addition, results from correlation and multiple linear regression analysis suggested that high levels of serum FGF21 were associated with age, obesity, and TG, which is in agreement with previously published studies [14, 20, 21]. Insulin dose was positively and significantly related to circulating FGF21 levels. This suggests that pancreatic beta-cell failure lowers basal FGF21 concentration and that FGF21 secretion may be maintained through insulin modulation in T1DM patients.

The liver is considered a major source of increased circulating FGF21 [1], but it is also secreted by skeletal muscle [22] and brown adipose tissue [23]. Thus, circulating FGF21 concentration may be affected by secretion from multiple organs. Chronic high levels of circulating FGF21 are considered as FGF21 resistant state [24], similar to insulin resistance, and is a risk factor for type 2 diabetes [15, 25] and cardiovascular diseases [21]. A lower basal insulin dose is associated with altered glycemic control in T1DM patients [26], and a chronically high dose of insulin can possibly cause FGF21 resistance, thereby exacerbating glucose metabolism and diabetic complications in patients with T1DM. These previous studies suggest that it is necessary to distinguish between beneficial FGF21 effects and the resistant state, and the determinant factors of circulating FGF21 have important implications for metabolic disease prediction and prevention.

There was no statistical difference with respect to age, BMI, lipid profile, and hepatic damage indices between the groups. However, T1DM patients showed lower serum FGF21 levels than non-diabetic individuals. Our findings are in agreement with previous animal studies and with the small sample sizes considered accordingly [7–10, 27]. It was reported that FGF21 is highly expressed in the exocrine pancreas [6], which may be affected by pancreatic beta-cell failure. The underlying mechanism may also be regulated by the FGF21 cleaving enzyme, serine dipeptidase fibroblast activating protein [10]. Although our study did not distinguish between FGF21 secretion from the pancreas and other organs, the interquartile range and median of serum FGF21 differed by approximately 55–90 pg/mL between T1DM and non-diabetic patients. This result is consistent with previously reported median difference of 87.7 pg/mL [8] and 75.8 pg/mL [9], and thus it is considered that basal FGF21 secretion from pancreas may be approximately one third of the circulating FGF21 level in middle-aged and elderly individuals [8, 11, 16, 25, 28–30]. On the other hand, it was reported that circulating FGF21 levels were more than 25-fold higher in hepatic steatosis (mean ± SD, 7.7 ± 2.9 ng/mL) as compared to healthy adults (0.3 ± 0.1 ng/mL) [30] and the frequency of undetected or abnormal levels of serum FGF21 did not differ between the groups in the present study. Therefore, the effects of pancreatic dysfunction on basal FGF21 secretion are smaller than those of hepatic dysfunction, which leads to an increase in circulating FGF21 concentration.

It has been considered that reduced FGF21 secretion impairs glycemic control in T1DM patients; hence, this study compared metabolic differences between T1DM patients with diabetic complications and those with non-diabetic complications. This study showed that T1DM patients with diabetic complications displayed poor glycemic control and dysfunction of liver and kidney tissues when compared with T1DM patients with non-diabetic complications. However, serum FGF21 levels were not associated with any diabetic complications. The findings of this study provide evidence that basal FGF21 levels may not play a key role in the progression of diabetic complications in middle-aged and elderly patients with T1DM.

Our correlation analysis showed a significant correlation between serum FGF21 levels and health-associated variables such as BMI, SBP, and TG after adjusting for age and sex. The association of serum FGF21 with BMI and TG is consistent with previous studies in T1DM patients, as described above. Although T1DM patients with diabetic complications were significantly older than T1DM patients with non-diabetic complications, there was no significant difference in BMI, SBP, and TG between the groups. It is likely that this lack of difference in these correlated variables is partly associated with similar serum FGF21 levels in T1DM patients with and without diabetic complications. Since it was reported that blood pressure and basal serum FGF21 levels are reduced by endurance exercise [16, 28, 29, 31], the relationship between SBP and FGF21 may reflect fitness level and physical activity in middle-aged and elderly patients.

To our knowledge, the present study is the first to demonstrate that insulin dose is positively associated with serum FGF21 levels in patients with T1DM. These results suggest that FGF21 secretion is stimulated by exogenous insulin, regardless of pancreatic dysfunction, as seen in healthy subjects [12]. In addition, our multiple regression analysis revealed that whereas aging and BMI were associated with higher serum FGF21 concentration, which is in agreement with previous studies [14, 20, 30], basal insulin dose was a determinant factor of serum FGF21 level in T1DM patients. This analysis revealed that the basal insulin dose was more effective than the bolus insulin dose for resting FGF21 levels. Since chronic high levels of blood FGF21 are a risk factor for several metabolic diseases [15, 21, 25], it is necessary to evaluate whether insulin dose simply affects FGF21 secretion and/or FGF21-related metabolic diseases. Moreover, the relationship between serum FGF21 levels and BMI suggests that obesity-induced FGF21 resistance may occur in T1DM patients. A 5 kg/m^2^ increase in BMI may correspond to approximately twofold increase in serum FGF21 levels, as calculated by the regression equation (S1 Table), suggesting that the chronic high level of blood FGF21 may have adverse effects on metabolic disease risk, as seen in healthy subjects [15, 21, 25]. Further studies are therefore required to study the effects of FGF21 resistance in T1DM patients.

Although this study evaluated the factors associated with circulating FGF21 in patients with T1DM, the cross-sectional study did not establish a causal relationship between FGF21 secretion and insulin dose. Moreover, blood samples were collected in clinical practice, which did not examine effects of consumption or skipping of breakfast in patients. It has been reported that FGF21 secretion is induced by nutrients such as low protein diet [32], fructose [33], soy protein [34, 35]; hence, there is a possibility that this nutrient intake affects circulating FGF21 levels in this study. It is necessary to examine the extent to which these nutrients induce FGF21 secretion in T1DM patients. FGF21 secretion has a diurnal rhythm [36]; thus, chronological and dose-dependent manors will provide more precise information. FGF21 levels did not increase from the morning to the afternoon [36], and 7 days of fasting may be required to increase serum FGF21 concentration [37]. The results suggest that the effects of sampling time and fasting status on serum FGF21 levels were not considered in our study.

In conclusion, circulating FGF21 levels are lower in patients with T1DM, suggesting that basal FGF21 concentration may be affected by pancreatic FGF21 secretion. No diabetic complications were related to serum FGF21 concentrations in Japanese middle-aged and elderly patients with T1DM. Moreover, serum FGF21 levels were also associated with aging and obesity, even in T1DM patients. Since insulin doses promote circulating FGF21, it is likely that pancreatic beta-cell failure does not impair insulin-stimulated FGF21 secretion. The beneficial effects of FGF21 on metabolic health are well recognized; thus, in the future, it is important to evaluate whether FGF21 inducible approach utilizing nutrients and/or pharmaceutical agents is beneficial for the prevention of diabetic complications and metabolic diseases in T1DM patients.

## Supporting information

S1 Table Relationship between BMI and estimated serum FGF21 levels in T1DM patients. The estimated serum FGF21 levels were calculated using stepwise multiple linear regression analysis (Table 4). ⊿FGF21 was calculated by subtracting the estimated serum FGF21 level with a BMI of 20 kg/m^2^. Regression equation: log FGF21 (pg/mL) = 0.500 + 0.032BMI (kg/m^2^) + 0.009age (years) + 0.012basal insulin dose (IU/day). We substituted 60 for age and 10 for basal insulin dose in the equation.
(PDF)

S2 Table Minimal data set. (XLSX)
