# Supplementary material for: Association of serum fibroblast growth factor 21 with diabetic complications and insulin dose in patients with type 1 diabetes mellitus
Source: PLoS One. 2022 Feb 22;17(2):e0263774. doi: 10.1371/journal.pone.0263774 (PMC8863253; doi:10.1371/journal.pone.0263774)
Supplement: S1 Table — The estimated serum FGF21 levels were calculated using stepwise multiple linear regression analysis (Table 4). ⊿FGF21 was calculated by subtracting the estimated serum FGF21 level with a BMI of 20 kg/m2. Regression equation: log FGF21 (pg/mL) = 0.500 + 0.032BMI (kg/m2) + 0.009age (years) + 0.012basal insulin dose (IU/day). We substituted 60 for age and 10 for basal insulin dose in the equation. (PDF) [file pone.0263774.s001.pdf]

S1 Table. Relationship between BMI and Estimated Serum FGF21 Levels in T1DM Patients

BMI, kg/m^2	Estimated FGF21, pg/mL	⊿ Estimated FGF21, pg/mL
20	758.6	
25	1096.5	337.9
30	1584.9	826.3
35	2290.9	1532.3

The estimated serum FGF21 levels were calculated using stepwise multiple linear regression analysis (Table 4). ⊿FGF21 was calculated by subtracting the estimated estimated serum FGF21 level with BMI 20 kg/m^2. Regression equation; log FGF21 (pg/mL) = $0.500 + 0.032_{\text{BMI (kg/m}^2\text{)}} + 0.009_{\text{age (year)}} + 0.012_{\text{basal insulin dose (IU/day)}}$.We substituted 60 for age and 10 for basal insulin dose in the equation.
